# Design and Implementation of an Electronic Patient Record via Adaptation of Existing Hospital Software

**DOI:** 10.7759/cureus.87763

**Published:** 2025-07-12

**Authors:** Tom Hebden, Emyr Jones, Robert Allcock, Badrinathan Chandrasekaran, Steve Ramcharitar

**Affiliations:** 1 General Medicine, Great Western Hospitals NHS Foundation Trust, Swindon, GBR; 2 Respiratory Medicine, Great Western Hospitals NHS Foundation Trust, Swindon, GBR; 3 Cardiology, Great Western Hospitals NHS Foundation Trust, Swindon, GBR

**Keywords:** digital information systems, discharge process efficiency, electronic patient record, human factors and ergonomics (hfe), quality improvement project

## Abstract

With the aim of improving efficiency and addressing limitations in paper-based clinical documentation, two resident doctors developed a customised electronic system for clinical note documentation on hospital wards. Using iterative co-design and active engagement with clinical colleagues, a ward moved from paper-based ward note-taking to electronic documentation. To demonstrate the benefits, the team conducted a comparative trial, assessing the in-house developed electronic patient record (EPR) system against the traditional paper notes in terms of efficiency and impact on working conditions. Following validation, the results were presented to, and endorsed by, the specialty Clinical Governance team. An implementation plan was subsequently formulated to introduce the EPR system across multiple hospital wards, utilising a stepwise approach. Various challenges emerged, leading to further iterative refinements to address issues encountered during each rollout phase. Challenges included raising hospital-wide awareness of participating wards, distributing training materials via channels compatible with real-world work patterns, overcoming hesitation about transition from paper-based systems, and addressing logistical hurdles with IT hardware deployment, which impaired the efficiency gains of the EPR system. Despite these challenges, the intervention demonstrated significant improvement in the timely handover of care at discharge from the hospital, with delays beyond 72 hours being eliminated.

## Introduction

Problem

The study was performed at a medium-sized district general hospital in the United Kingdom (UK). At present, only 20% of National Health Service (NHS) organisations are digitally mature, although 86% have a form of electronic patient record in place [1]. The NHS England initiative ‘What Good Looks Like’ (WGLL) and the national target of achieving a ‘digitised frontline’ by 2025 indicate a top-down push toward integrated electronic patient records (EPRs). However, adoption is patchy due to funding, infrastructure constraints, differing procurement processes, and cultural resistance.

At the commencement of the project, paper documentation remained the primary method for recording clinical notes across most hospital departments, with the exception of the emergency department, intensive care unit, and obstetrics, each of which employed separate and non-integrated EPR systems. The earlier adoption of electronic systems in these specialties likely reflects a combination of departmental prioritisation, access to targeted funding streams, and the high-acuity nature of care in these settings, which may have strengthened the case for earlier digitisation.

Electronic systems were deployed for core clinical functions, for example, electronic prescribing and medicine administration (EPMA), Order Comms (Clinisys ICE), picture archiving and communication systems (PACS), electronic observations (eObs), digital radiology via PACS, and other specialised programs for specific fields. Clinicians on ward rounds recorded their work using paper. Additionally, they are required to gather and integrate information from several electronic systems to create an accurate and up-to-date patient profile each day.

This dual-modality process contributes to mental fatigue, increases the frequency of errors, and consumes time in collating information efficiently, resulting in an extended length of stay and delays to performing important tasks such as completing handover letters at discharge.

Available knowledge

Paper-based patient records have long been the standard in healthcare, valued for their familiarity among senior clinicians and the tangible, chronological narrative they offer. Paper is easy to annotate and requires no specialised hardware whilst remaining functional during power outages or system failures [2].

However, paper records have significant limitations. Issues with legibility, difficulties in sharing information, and risks of data loss or misplacement are common. Storage and retrieval are cumbersome, particularly in high-volume settings, and delays in digitising records through systems such as electronic document and records management systems (EDRMS) can hinder timely access. For clinicians, manual documentation is physically taxing, inefficient, and prone to duplication of effort [3]. The lack of automated backup systems also leaves paper records vulnerable to permanent loss. These drawbacks have driven the shift towards EPR systems, which address many of these inefficiencies. However, widespread adoption in the NHS remains challenging due to the high upfront costs of hardware and ongoing support requirements, which can strain hospital budgets. Furthermore, complexities exist at sociocultural and sociotechnical levels, with Greenhalgh et al. describing complex interdependencies, inherent tensions, and high implementation workload [4] that must be considered in EPR transitions.

Rationale

All healthcare systems face the challenge of increasing demands for services caused by aging populations, improved survival with complex conditions, and increasing needs of patients with complex long-term conditions. At the same time, all economies are struggling to fund their health systems, with the gap between available funding and the costs of desired care widening. At a hospital level, there is an escalating need to find ways to reduce the cost of providing care, to enable clinicians to add more value whilst using less time, and to eliminate things that cause delays and inefficiencies.

EPR systems are costly and often locked into long-term contracts, limiting a hospital’s ability to transition to more optimal solutions [5]. Currently, this hospital utilises multiple systems. Through a process of trial and error, it was discovered that an existing system used for nursing observations and tasks could be adapted for clinical documentation by clinicians. This adaptation would also allow allied healthcare professionals (AHPs) to document their findings, thereby creating a unified system for all professionals within clinical environments. Such a unified, electronic system would enhance collaborative information sharing, which was often compromised under the previous paper-based system.

Given the costs and challenges associated with deploying new systems, adapting the already-deployed software presented a unique opportunity. This approach would eliminate the need for substantial upfront capital expenditure, creating a critical advantage for the hospital. This prospect provided strong momentum for modifying the software (NerveCentre™) to support electronic documentation by clinicians.

Specific aims

This project encompassed the complete process of deploying an EPR system, starting with iterative modifications to adapt pre-existing software. The implementation began with a trial phase, during which the system was used alongside traditional paper documentation. Findings from this phase were reviewed and scrutinised by the hospital’s Digital Transformation Board (DTB) before progressing to a structured, phased rollout with ongoing support across multiple departments.

The primary objectives were to enhance documentation efficiency for clinicians, improve the timely completion of electronic discharge summaries (EDS), and alleviate the mental and physical demands associated with handwritten notes, ultimately improving the quality of working conditions.

## Materials and methods

Context

The hospital already employed the NerveCentre™ software for nursing observations and task management. This project's unique approach involved adapting the pre-existing system to accommodate clinical documentation, thereby circumventing the financial and logistical challenges of deploying entirely new software.

Effective patient care during the transition from hospital to home hinges on the seamless handover of information to general practitioners (GPs). Upon discharge, it is imperative that GPs receive comprehensive details, including the primary diagnosis leading to admission, a succinct summary of the patient's medical history, any clarifications made during the hospital stay, adjustments to medications, and recommendations for follow-up tests or imaging to monitor recovery. Such thorough communication ensures continuity of care, reduces the risk of readmission, and enhances patient outcomes [6].

In many UK hospitals, the responsibility of crafting discharge summaries falls to resident doctors. However, due to time constraints and less-than-optimal systems, there have been instances where patients are discharged without timely or complete handover documentation. Addressing this challenge is paramount, as delays or omissions in discharge summaries can compromise patient safety and impede the effectiveness of subsequent care [7].

Intervention

The intervention involved adapting the existing NerveCentre™ system - already in use for electronic observations and nursing assessments - to support real-time clinical note-taking by doctors and AHPs. The system offers a structured free-text interface under predefined headings, including ‘Reason for admission ’, ‘Diagnosis’ (current working diagnosis ), ‘Past medical history’ (medical history and problem list), ‘Doctor note ’, and ‘Clinical plan’.

The design process aimed to balance universality across all specialties with the flexibility to accommodate specialty-specific requirements. Early iterations included additional headings such as ‘Investigations’ and ‘Blood Tests’; however, these were found to fragment information due to the system's non-shared architecture. Through iterative development, it became evident that structuring data fields around the patient, rather than individual specialties, provided a more cohesive patient portrait accessible to all clinicians. Initially, a ‘Clinical plan’ field was incorporated to record concise plans. However, this led to the plan and its rationale becoming disconnected, posing potential risks. Consequently, this field was removed to maintain the integrity of clinical documentation. This approach fosters a continuous handover model within ward notes, reflecting modern team-based medical practice where different doctors may see a patient each day. The ‘Doctor note’ section serves as a flexible space for specialty-specific details arising during clinical encounters. The iterative design process, conducted over several months, ensured the software was optimised for multidisciplinary use while aligning with existing hospital policies and workflows.

While NerveCentre™ was not initially deployed as a comprehensive EPR system, its structure facilitated agile and immediate completion of discharge handover letters. Despite the absence of formal integration between NerveCentre™ and the EDS system, clinical notes from NerveCentre™ could be efficiently transferred into the EDS with minimal editing, enabling prompt communication of discharge information to GPs.

Furthermore, content recorded in NerveCentre™ is immediately available to clinicians upon patient readmission. The ‘Past medical history’ field persists between inpatient spells, allowing clinicians to resume care seamlessly using detailed information from prior encounters.

The free-text nature of NerveCentre™ fields enables clinicians to document granular details of clinical problems. The significance of a comprehensive clinical problem list has been recognized since Lawrence Weed introduced the problem-oriented medical record in the 1960 [8]. Some EPR systems have shifted towards coded categories for diagnoses, primarily for financial reimbursement, which can obscure detailed problem lists and contribute to clinician frustration. This project successfully allowed clinicians to record information in a manner best suited to support efficient patient care.

The system rollout commenced on pilot wards and was underpinned by iterative feedback loops designed to refine training materials, respond to user concerns, and resolve practical issues related to hardware availability. Staff were introduced to the new system through dedicated training sessions, and support was made readily accessible by embedding project leads within daily ward teams throughout the transition period. Ongoing improvements were guided by regular meetings and user feedback surveys, whilst supplementary online resources such as intranet-based training modules and instructional videos were made available to reinforce learning. To support efficient working practices, additional computer workstations were installed on wards, equipped with agile tap-in login functionality to streamline access. This structured and responsive implementation strategy aimed to smooth the transition and helped promote both widespread adoption and effective utilisation of the NerveCentre™ system.

Study and measures of the intervention

The study aimed to evaluate the efficacy of NerveCentre™ as a replacement for paper-based systems. Data collection included pre- and post-implementation surveys, which assessed subjective user experiences and quantitative metrics, including timely completion of a discharge handover letter to the GPs when patients leave hospital. The project also reviewed qualitative feedback on efficiency, thoroughness of documentation, and the perceived impact on inpatient care on wards.

Key areas of focus in the project included the time required to complete EDS before and after the intervention, with particular attention to completion rates within 24, 48, and 72 hours. The evaluation also explored user preferences regarding paper versus electronic systems, providing insight into usability and acceptance across different clinical roles. Additionally, the project examined the impact of the digital system on ward round preparation and the perceived quality of documentation. This was assessed in terms of how effectively the records supported handover and enabled the next shift doctor to deliver optimal patient care.

Analysis

Statistical analyses, including t-tests, p-values, and Cohen's d calculations, were used to compare EDS completion rates pre- and post-implementation of the intervention. Graphical representations highlighted trends in EDS completion rates, whilst tables displayed the preferences for digital documentation among resident clinicians versus consultants.

EDS data were sought from central operations that provided EDS completion rates retrospectively. A 12-month pre- and post-intervention was chosen. Ward A was chosen as this was the ward that had a 12-month pre- and post-intervention data available (other wards did not have sufficient data due to the staggered roll-out approach). Percentages of EDS completion rates reflect EDS completion for all patient discharges from Ward A for any given month. Frequency (n) / raw data were not possible to access from central operations (Trust Informatics Report) for EDS completion rates.

The analysis employed three key metrics to evaluate the completion rates of EDS across all four timeframes (up to 24 hours, up to 48 hours, up to 72 hours, and over 72 hours) following implementation of the intervention on Ward A. The analysis covered May 2022 to June 2024, with the intervention implemented from May 2023 onwards. Statistical analysis and data visualisation were performed using Python on Google Colab (a cloud-based Jupyter Notebook environment). Welch’s t-test was employed to compare means, as it appropriately accommodates unequal variances between groups. Statistical significance was assessed using p-values, with a conventional threshold of p < 0.05 (α = 0.05) indicating significance. To quantify the magnitude of improvement, Cohen’s d was calculated as a measure of effect size. This metric helps interpret the practical significance of results, where a value between 0.2 and 0.5 is considered a small effect, between 0.5 and 0.8 a medium effect, and any value greater than 0.8 is interpreted as a large effect.

Ethical considerations

The project involved several ethical considerations regarding awareness and mitigation of potential risks to patient and staff safety associated with systematic changes to ward operations. To address these concerns, a risk strategy meeting was convened with the DTB and senior clinicians. Clinical Safety Officer advice was sought, consistent with DCB 0160. A hazard workshop model meeting and discussions identified potential risks and agreed safeguards to mitigate them, ensuring the safety and well-being of both patients and staff throughout the implementation process.

The rollout was conducted on a semi-voluntary basis, where wards were identified as suitable for initial implementation based on clinician appetite. Expansion was then pursued after a thorough review of safety and operational readiness, accompanied by clear communication with staff through email, ward visits, and Trust-wide communications. As wards adopted the new way of working, other clinical teams noted their success, and resident doctors rotating between wards contributed to spreading the desire to move away from using paper records.

The optional anonymous survey (broadcast by Trust Communications to all members of staff in the hospital) gave healthcare practitioners the opportunity to express their opinions on the intervention. This survey did not require ethics committee approval or Institutional Review Board (IRB) review, as participation in the survey was voluntary and anonymous, and there was no incentive offered for completion. The Trust agreed that the survey was in the scope of Trust Communications and so no further approval was gained.

The authors declare no conflicts of interest with the project.

## Results

**Figure 1:**
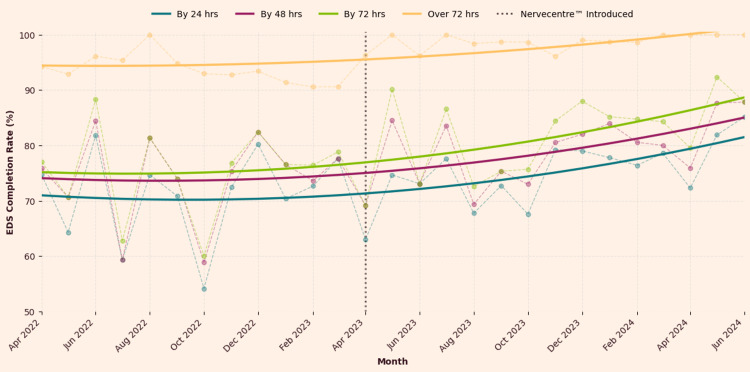
EDS completion rates (March 2022-July 2024) EDS: Electronic discharge summaries

The line graph (Figure 1) illustrates a clear upward trend in the proportion of EDS completed within 24 and 48 hours, whilst delays over 72 hours were entirely eliminated.

**Figure 2:**
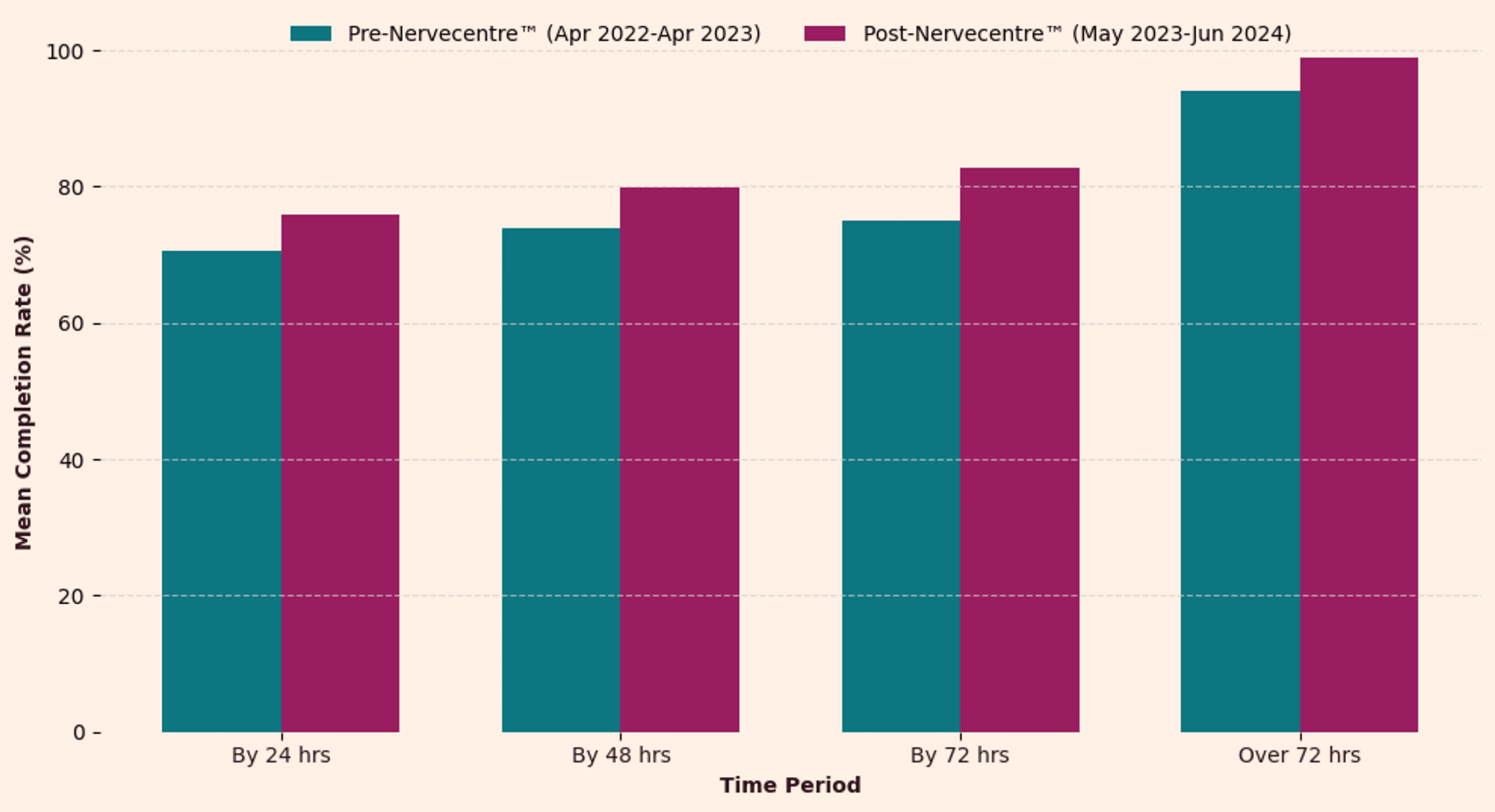
Comparison of EDS completion rates: pre vs. post Nervecentre™ EDS: Electronic discharge summaries

The bar chart (Figure 2) compares the mean EDS completion rates before (April 2022-April 2023) and after (May 2023-June 2024) the implementation of the NerveCentre™ EPR system across the four timeframes.

Tables 1-2 display a summary of the 57 responses that were gained from the implementation survey.

**Table 1:** User perceptions of Nervecentre™ vs. paper documentation

Question	Inferior to paper (%)	Equal to paper (%)	Superior to paper (%)
Using Nervecentre™ for discharge summaries	17.02	19.15	63.83
Prepping patient notes for ward rounds	21.15	21.15	57.69
Thoroughness of electronic documentation	35.29	27.45	37.25
Efficiency: handwriting vs typing	17.31	7.69	75.00
Morning board-round efficiency	16.98	22.64	62.26
Paperless ward rounds experience	38.46	5.77	55.77

**Table 2:** Preference for paper vs digital documentation by grade AHPs: Allied health professions; IMT: Internal medicine training

Grade	Prefer paper (%)	No preference (%)	Prefer digital (%)
Foundation	17.39	8.70	73.91
IMT/CT	45.45	9.09	45.45
Registrar/SAS	20.00	0.00	80.00
Consultant	81.82	0.00	18.18
Other (AHP/nurse)	50.00	0.00	50.00

Table 3 presents a comprehensive statistical comparison of EDS completion rates before and after the implementation of the NerveCentre™ EPR system.

**Table 3:** Statistical analysis of EDS completion rates: pre vs. post Nervecentre™ EDS: Electronic discharge summaries

Time period	Mean (Pre)	Mean (Post)	Welch's t	p-value	Cohen's d
By 24 hrs	70.47%	75.98%	2.09	0.05	0.82
By 48 hrs	73.77%	79.80%	2.27	0.03	0.89
By 72 hrs	74.94%	82.84%	2.86	0.01	1.11
Over 72 hrs	93.94%	98.88%	6.09	0.00	2.40

Interpretation

The statistical analysis demonstrates a significant improvement in the timely completion of EDS following the implementation of the NerveCentre™ EPR system. Key statistical measures (Welch’s t-test, p-values, and Cohen’s d) were employed to compare pre- and post-intervention EDS completion rates across four timeframes: within 24 hours, 48 hours, 72 hours, and beyond 72 hours.

The analysis revealed a progressive improvement in mean completion rates following the intervention, with statistically and clinically meaningful differences observed across all timeframes. Within 24 hours, the average completion rate rose from 70.47% to 75.98%. This change approached statistical significance, with Welch’s t = 2.09 and a p-value of 0.05. The associated effect size, Cohen’s d = 0.82, suggested a large practical impact. At 48 hours, mean completion improved from 73.77% to 79.80%. This difference reached statistical significance (t = 2.27, p = 0.03), with a similarly large effect size (d = 0.89), reinforcing the intervention’s clinical relevance. By 72 hours, the completion rate increased from 74.94% to 82.84%, a difference that was highly statistically significant (t = 2.86, p = 0.01). The effect size here was very large (d = 1.11), highlighting the intervention’s strong efficacy in reducing delays. Beyond 72 hours, completion rates approached universality, increasing from 93.94% to 98.88%. This improvement was extremely statistically significant (t = 6.09, p < 0.00001), and the corresponding effect size (d = 2.40) was exceptional, indicating that the intervention had virtually eliminated prolonged discharge summary delays and significantly improved the timeliness of handover to primary care.

A pre- and post-implementation survey was conducted to subjectively assess the impact of the intervention. This hospital-wide survey, disseminated via internal communications, garnered responses from 57 participants (consultant = 11, foundation doctor (F1/F2) = 23, CT/IMT/SHO = 11, registrar/SpR = 7, senior charge nurse = 1, ward sister = 1, unknown = 3). Survey questions were not mandatory, so total responses for each question varied.

The findings, detailed in Tables 1-2, reflected an overall preference for digital documentation, with 63.80% (n = 30/47) of respondents rating the new system as superior to paper-based discharge summaries, whilst only 17.02% (n = 8/47) viewed it as inferior. Additionally, 75.00% (n = 39/52) of participants reported greater efficiency when typing notes rather than handwriting them, suggesting that the digital platform not only streamlined workflow but also alleviated some of the physical burden associated with manual documentation. In terms of clinical utility, 57.69% (n = 30/52) of clinicians expressed a preference for conducting ward rounds without paper, and 62.26% (n = 33/53) observed improved efficiency and communication during board rounds. These results suggest that real-time access to electronic patient data enhanced team interactions and supported more informed decision-making. However, adaptation to the digital system varied notably by seniority. Foundation doctors showed the strongest preference for the new approach, with 73.91% (n = 17/23) favouring it over paper, whereas consultants were markedly less enthusiastic, with 81.82% (n = 9/11) still preferring traditional methods. This divergence may reflect generational differences in technological fluency, with junior staff more adaptable to digital workflows, whilst senior clinicians may be constrained by steeper learning curves or long-standing habits. Despite these positive trends, only 37.25% (n = 19/51) of respondents felt that their documentation became more thorough post-intervention, indicating that there may be a need to refine system design or enhance training to better support comprehensive and high-quality clinical record-keeping.

## Discussion

The implementation of NerveCentre™ as an EPR system demonstrated measurable improvements in efficiency and documentation accuracy, particularly among junior clinicians. These findings align with Clarke et al.’s observations, where interviewees highlighted the anticipated benefits of EPR implementation, including improved efficiency, availability, and accessibility of clinical information, as well as enhanced patient safety [3]. Whilst these positive outcomes reflect the potential of EPRs to streamline secondary workflows, such as audit and discharge documentation, they also highlight a tension in the literature: primary clinical work may become less efficient despite gains in administrative tasks [2]. For instance, although real-time data entry reduced delays in EDS completion, the system’s rigid structure, contrasted with paper’s unique adaptability, may still impose burdens on dynamic clinical ward-based workflows as reflected in our survey.

The slower adoption among senior staff revealed in our survey reflects a broader challenge in EPR implementation: balancing the conceptualisation of users as 'information-processors' with their role as members of a 'socio-technical network' [2]. This dynamic highlights the need to address not only individual perceptions or skill gaps but also the wider social and organisational dynamics that shape how EPRs are used and integrated into clinical workflows. Resistance to change in this context aligns with Kahneman’s dual-process theory, which suggests that clinicians, particularly those with established workflows such as consultants, operate predominantly in System I thinking - a fast, intuitive mode based on habit and experience [9-10]. The transition to electronic documentation requires engaging System II thinking, a slower, effortful process, which is naturally resisted even when the benefits of change are evident. Additionally, loss aversion, where potential losses are psychologically weighed more heavily than equivalent gains, may contribute to initial reluctance as clinicians perceive the shift as a disruption rather than an improvement [10]. Our iterative refinement approach acknowledged this duality, combining technical functionality with human input, using feedback loops to adapt at each stage of the roll-out - a critical factor for implementation success. Our survey findings mirror this sentiment, reinforcing that success is not merely a technical achievement but a socially negotiated process. Cultural uncertainty, as highlighted by Rivard et al. [11], and infrastructure demands [12] were key barriers, reinforcing that EPR adoption involves a reconfiguration of organisational context from the setting of implementation to the EPR-in-use. This socio-technical theory also resonates with Clarke et al.’s emphasis on the interplay between technical and social factors in EPR adoption [3].

By repurposing an existing system for clinical documentation, the project bypassed the established statutory processes, such as those outlined in DCB0129/DCB0160, that govern safety and hazard management in healthcare technology transfers. Typically, when a supplier hands over a product, it undergoes rigorous hazard analysis and the formulation of a safety case to identify and mitigate potential risks before implementation. However, adapting software that was originally deployed for another clinical function may mean that hazard controls or safety cases are not designed into the system from the outset. This reactive approach to identifying issues (e.g., duplicate entries were a common issue raised by clinicians interacting with Nervecentre™ due to the inherent nature of the software architecture, which prevents appended entries to clinical notes) can introduce unforeseen risks. These challenges are consistent with the findings of Sittig et al. [13], who advocate for a sociotechnical approach that integrates safety controls throughout the lifecycle of health information technology. Similarly, Reason’s [14] work on human error suggests that, although deliberate (System II) thinking is generally more analytical and tends to reduce errors, the sudden change from familiar, automatic (System I) processes to relying on conscious, slower reasoning can impose a significant cognitive burden. In situations where there is time pressure or fatigue, this increased load may temporarily expose clinicians to a higher risk of error as they adjust to the unfamiliar workflow [14].

Alongside technical risks, this project has highlighted the importance of adequate project infrastructure and resource allocation. Despite the enthusiastic effort by two resident doctors, the initiative suffered from an absence of robust project management support that would have been necessary for full-scale deployment. Incomplete deployment may lead to challenges, such as inconsistencies in system use and additional safety risks, if sufficient support is not provided. Evidence from studies by Carayon et al. suggests that aligning technology, tasks, and organisational factors is critical for patient safety and overall system effectiveness [15]. Debates over deployment strategies, whether opting for a big bang approach or a piecemeal roll-out, reflect a trade-off between rapid transformation and the need for iterative refinement based on end-user feedback. On the one hand, a big bang deployment might streamline the transition by abruptly eliminating legacy systems. However, it can also overwhelm users who must adapt to new workflows without sufficient time for proper acclimatisation. Ratwani et al. [16] highlighted that rapid, large-scale implementations are more prone to exposing usability challenges, which can lead to increased error rates and hinder user acceptance because clinicians face a steep learning curve when forced to quickly adapt to unfamiliar interfaces and disrupted routines. Conversely, a piecemeal roll-out, such as the approach adopted in this project, offers the advantage of incorporating iterative adjustments. By allowing users to provide feedback throughout the implementation process, the system can be fine-tuned incrementally, resulting in a more adaptable and ultimately robust solution. Although this method may lead to prolonged periods of hybrid operations where legacy and new systems coexist, which can cause workflow inconsistencies and compounded transitional hazards, it facilitates a smoother transition and provides targeted support for varied user groups. Campbell et al. have demonstrated that, without careful alignment of clinical context and technical infrastructure, even well-intentioned system implementations can introduce new error modes [17]. Thus, whilst a phased approach may extend the transitional period, it also enables continuous improvement and a more resilient system design that better supports clinical workflows.

The strategy of repurposing existing software rather than pursuing large-scale systems aligns with evidence that smaller systems may sometimes be more efficient and effective, particularly in resource-limited settings [2]. This strategy stands in interesting contrast to Kaiser Permanente’s experience, where Scott et al. documented significant challenges with software development costs and vendor relationships [5]. By repurposing existing software, we circumvented many of these financial and development hurdles, though the successful implementation still required overcoming cultural hesitancy, as explored by Rivard et al. [11], and ensuring adequate technical infrastructure, including the availability of sufficient terminals [12]. While confounding factors such as workforce changes, additional locum shifts, and hospital-wide efforts to reduce EDS backlogs may have influenced our outcomes, the results suggest that hybrid approaches, blending EPR efficiency with paper-like adaptability, could optimise clinical workflows.

Future research should explore how smaller, flexible EPR systems might sustain efficiency without compromising clinical practice, particularly in resource-constrained environments.

Limitations and considerations

The project faced several limitations that impacted the rollout and adoption of the NerveCentre™ EPR system. Insights into these challenges were largely drawn from the optional ‘free text’ feedback provided anonymously during the post-implementation survey, which offered valuable perspectives on the user experience. 

Ergonomic factors, such as the placement of fixed terminals, impacted ease of use during ward rounds. Human factors, including variability in user engagement and adaptability, highlighted the need for tailored communication and stronger leadership support across departments. Resistance to EPR implementation has been widely recognised in healthcare settings, and studies have emphasised the importance of stakeholder engagement and robust support systems in overcoming this challenge [2-4,18]. Training accessibility posed another consideration, particularly for staff on irregular shifts, emphasising the importance of flexible and widely available learning resources. Streamlined feedback mechanisms could further enhance user experience by accelerating refinements based on real-world usage.

Hardware availability and reliability emerged as areas for improvement, with portable device shortages and connectivity issues affecting workflow efficiency. Similar challenges have been highlighted in previous studies, where technical infrastructure issues, including hardware shortages and network reliability, were identified as key barriers to effective EPR implementation [19].

## Conclusions

This project highlights the potential for substantial efficiency gains through the creative adaptation of existing software platforms, delivering the benefits of digitisation without the need for new procurement, extensive contracts, or major infrastructure changes. By leveraging systems already familiar to staff, we implemented a low-cost, agile solution well-suited to resource-constrained settings. A phased rollout, underpinned by continuous feedback loops, proved critical in managing change and addressing challenges in real time. This approach offered a viable alternative to traditional large-scale EPR deployments and was particularly effective given the project’s reliance on a small, non-funded team. Future efforts should focus on refining hardware integration and engaging resistant demographics.

## Appendices

Notes

The project included a structured hazard assessment process, documented in a formal hazard log reviewed by the Clinical Safety Officer in line with DCB0160 requirements. Several key patient and staff safety concerns were identified:

Hybrid documentation risk: During the phased rollout, not all patients had their records on NerveCentre, which could lead to confusion about where the most up-to-date information resided. To mitigate this, we introduced a yellow label system to visibly mark patient records in transition and provided clear guidance and training materials to all staff, including visiting teams.

Dual-platform risk: Patients transferred between wards with and without the new system could have incomplete or fragmented notes. This was addressed by requiring printed records to accompany patients and reinforcing awareness of the process through targeted communication.

Access and visibility risk: There was concern that clinicians accessing records after discharge or in other systems might be unaware of the location of key documentation. Additional training and targeted updates to smaller groups (e.g., Medical Examiners, Legal teams) were used to address this.

Duplicate entry risk: Because NerveCentre lacked structured templates or append-only functionality, there was potential for duplicate or conflicting entries if users updated entries rather than adding a new entry (as one would do in paper notes). This was mitigated through online training material and reinforcing a clear workflow for note creation.

Ergonomic and hardware limitations: Limited availability of workstations and fixed-terminal placement created challenges for clinicians to access and document care efficiently during ward rounds. These issues were partly addressed by deploying additional devices, though we recognise this remained an area for improvement.
